# The Impact of the COVID-19 Pandemic on Villages: Results of a National Survey

**DOI:** 10.1093/geroni/igaa057.3507

**Published:** 2020-12-16

**Authors:** Natalie Galucia, Nancy Morrow-Howell, Peter Sun, Tanner Meyer, Ying Li

**Affiliations:** 1 Washington University in St. Louis, St. Louis, Missouri, United States; 2 Washington University in St. Louis, Saint Louis, Missouri, United States; 3 Washington University, St. Louis, Missouri, United States

## Abstract

This study, launched in June 2020, documents the impact of the COVID-19 pandemic on Villages nationally. Villages are non-profit, membership-based organizations that provide support from volunteers and social connections to enable aging in place. We distributed on-line surveys to the leaders of the 287 Villages in the national network to capture the effects of the pandemic on organizational operations, membership recruitment, service provision, and member well-being. A 40% response rate (n=116) was obtained. A majority of Villages reported that the pandemic greatly affected the organization, with the top concerns being: 1) membership recruitment, 2) the health and well-being of members and volunteers and 3) connecting with their members outside of normal in-person events. Over half of the respondents reported that the mental health of members had declined; and there were high levels of disruption to usual health care. New member recruitment efforts were thwarted and most Villages lost revenue. About 70% offered virtual programming but, in general, participation in these on-line events dropped. From the survey respondents’ perspective, the value of the Village to members and their family increased (48%) or remained the same (22%). New opportunities emerged that may be continued post-pandemic: new meal and medicine delivery volunteer services, more on-line communication and telephone reassurance, and new family and community connections. Findings indicate a wide range of experiences during the pandemic, with variation stemming from age of the Village and size of membership. The study informs the sustainability and growth efforts of Villages during and after the pandemic.

